# Using Individual GPS Trajectories to Explore Foodscape Exposure: A Case Study in Beijing Metropolitan Area

**DOI:** 10.3390/ijerph15030405

**Published:** 2018-02-27

**Authors:** Qiujun Wei, Jiangfeng She, Shuhua Zhang, Jinsong Ma

**Affiliations:** Jiangsu Provincial Key Laboratory of Geographic Information Science and Technology, School of Geographic and Oceanographic Sciences, Nanjing University, Nanjing 210023, China; qiujun@smail.nju.edu.cn (Q.W.); zhangshuhua11@mails.ucas.ac.cn (S.Z.); majs@nju.edu.cn (J.M.)

**Keywords:** foodscape exposure, activity space, commuting route, space-time kernel density estimation, time-weighted exposure, Beijing

## Abstract

With the growing interest in studying the characteristics of people’s access to the food environment and its influence upon individual health, there has been a focus on assessing individual food exposure based on GPS trajectories. However, existing studies have largely focused on the overall activity space using short-period trajectories, which ignores the complexity of human movements and the heterogeneity of the spaces that are experienced by the individual over daily life schedules. In this study, we propose a novel framework to extract the exposure areas consisting of the localized activity spaces around daily life centers and non-motorized commuting routes from long-term GPS trajectories. The newly proposed framework is individual-specific and can incorporate the internal heterogeneity of individual activities (spatial extent, stay duration, and timing) in different places as well as the dynamics of the context. A pilot study of the GeoLife dataset suggests that there are significant variations in the magnitude as well as the composition of the food environment in different parts of the individual exposure area, and residential environment is not representative of the overall foodscape exposure.

## 1. Introduction

The relationship between the built environment and individual health has long been of interest to the public and researchers [1]. Within nutritional and epidemiological research, substantial focus has been placed on uncovering the spatial inequalities of the food environment (“foodscape”) and measuring their effects on personal health outcomes as well as eating behaviors such as obesity, body weight, body mass index (BMI), and food consumption [2,3,4,5,6]. Historically, most studies characterized the food environment solely based on the residential neighborhood including administrative units and residence-based buffers [7]. Such choices were primarily due to the availability of censuses and surveys data that can be easily used to estimate the population health [8]. However, the spatial extent of a neighborhood was individual-specific, and the artificially designated neighborhood often failed to coincide with the observations [9,10]. Indeed, people are not bound to their neighborhoods: they move around to perform their routine activities and may encounter different types and levels of resources in their activity locations [11].

The popularity of location-aware devices, geo-sensor networks, and web-based mapping tools enables us to objectively collect detailed data on human movement, which is a favorable step towards the refined assessment of environmental exposure accounting for daily mobility [12]. Recently, a growing body of studies have used GPS-based travel surveys to measure the food exposure and have made great progress in re-examining the effects of non-residential contexts in foodscape exposure [13,14,15]. For example, Christian et al. (2012) compared neighborhood-based food exposure with activity-based food exposure and found that 83.4% of the participants encountered very different food environment in their daily travels compared with the residential neighborhoods [16]. Conventionally, the majority of the current literature have generally used the short-termed GPS data and adopted the overall activity space—the subset of all the locations within which an individual has direct contact as a result of his or her day-to-day activities—to conceptualize the environment experienced by the individuals [17]. They employed exhaustive GPS logging points and generated a uniform geospatial boundary in the form of standard deviation ellipse (SDE), daily path area (DPA), kernel density surface or minimum convex polygon (MCP) to represent the exposure area [18,19,20]. However, this approach has some limitations.

The widely-used overall activity space ignores the internal heterogeneity of its component places and the complexity of daily movement [21,22]. Basically, human activities are multi-centered [23]. The daily life centers (also termed anchor points) are usually composed of the places where people organize their daily activities (e.g., home) and to which people are relatively obligated to go (e.g., workplace) [24]. Each anchor point serves an aspect of daily life for the individual (e.g., dwelling, working, schooling, and recreation) and in turn the activities around these places have different spatial patterns and temporal rhythms such as the spatial extent people move around, the activity duration and the timing of these activities, which are very relevant to quantify the individual exposure [8]. Nevertheless, the uniform spatial delimitation of the overall activity space usually involves a considerable part of unexperienced areas that the participant rarely visits and would indirectly give rise to the overestimation of individual exposure [25]. Moreover, this uncertainty is particularly evident in the short-period GPS studies, wherein the observation window of a few days could hardly capture the relevant geographic contexts that an individual encounter and may import some occasional travels in this period [26,27]. To address this issue, a promising solution is to differentiate GPS data based on the characteristics of the activities practiced at different places and reconstruct the multiple contexts from the long-term GPS data [28]. Despite the advance in theory, little progress has been made in practice to incorporate the spatial extent and temporal patterns of the individual activities into the foodscape exposure assessment in a multi-context environment from GPS data [29].

The overall activity space also ignores the heterogeneity of the transportation modes of individual commuting behavior [30]. To date, numerous GPS-based studies have simply utilized all the traveling paths to model the exposure areas and failed to account for the transportation modes [20,31,32]. However, people within high-speed vehicles or underground transport tend to isolate themselves from the outside environment, and therefore have less opportunity to access food outlets without the vehicle stopping and them getting out [30]. To this end, it will be more reasonable for researchers to investigate the individual foodscape exposure along the commuting journeys based on the non-motorized trips rather than the entire paths [24,30].

In this paper, we proposed investigating individual foodscape exposure from the long-term GPS trajectories using a novel framework to incorporate the localized activity spaces around the daily life centers (residence, workplace and other major places) and non-motorized commuting routes across these places (Figure 2). To address this issue, we first integrated the density-based spatial clustering of applications with noise (DBSCAN) and the space-time kernel density estimation (ST-KDE) to identify clusters of frequently visited places [33,34]. A supervised machine-learning method, namely, the stochastic gradient descent (SGD) classification, was later used to extract the non-motorized segments [35]. Then, the clusters of frequently visited places and non-motorized commuting routes were used to construct the exposure areas. Finally, a case study in the Beijing metropolitan area was conducted to explore the characteristics of food environment exposure around the residence, workplace, other major places, and along the commuting journeys from a long-time perspective.

## 2. Study Dataset

**Figure 1:**
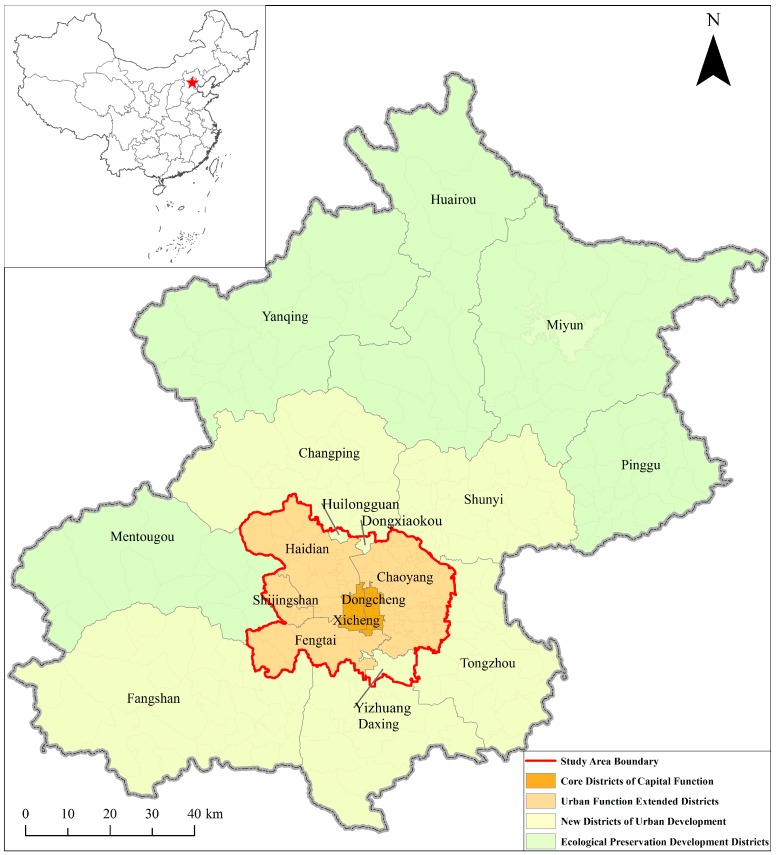
The study area consists of the urban area and three suburb districts with dense residential communities or industrial parks (Huilongguan, Dongxiaokou, and Yizhuang).

The study area consisted of the urban area and three suburb districts with dense residential communities or industrial parks (Huilongguan, Dongxiaokou and Yizhuang) in Beijing, with an area over 1473 km^2^ (Figure 1). The GPS dataset was part of the GeoLife project by Microsoft Research Asia (MSRA) in Beijing [36]. The preliminary dataset contained 17,621 GPS trajectories collected by 182 volunteers from a period of over five years (from April 2007 to August 2012). In the data collection program, a great portion of participants (*n* = 107) remained living in the study area, while some of the others only stayed in Beijing for a few months and then migrated from/to other cities later. The GPS loggers used in the project were handheld GPS receivers including Magellan Explorist 210/300, G-Rays 2 and QSTARZ BTQ-1000P. In general, the sampling rate of the GPS loggers was two seconds and the positioning accuracy was more than three meters. In this dataset, a GPS trajectory was a time-stamped sequence of GPS points $p_{i}=\left(x_{i},y_{i},t_{i}\right)$, where $x_{i},\;y_{i}$ and $t_{i}$ represented the latitude, longitude and time-stamp, respectively. Typically, each trajectory recorded one complete trip of individual movement in the outdoor space, such as going to work, going home or leisure activities, and a considerable part of the trajectories were annotated with transportation modes by the participants (9813 tracks from 73 users).

The points of interest (POIs) dataset were obtained from the AutoNavi Map, a Chinese navigation and location-based service provider. The dataset was collected in 2012 and was the only POI data available to us, which contained various types (22 groups, 728 classes) of POIs, such as the commercial facilities, hospitals, schools, and residential communities in Beijing. After online validations of the POIs, we classified the food services into five categories for exposure estimation, namely, convenience stores, fast food outlets, supermarkets, restaurants, and vegetable and fruit stores based on the Standard Industrial Code (SIC). Furthermore, the road and public transport datasets were supplied from the Beijing Municipal Commission of Transportation [37].

## 3. Methodology

**Figure 2:**
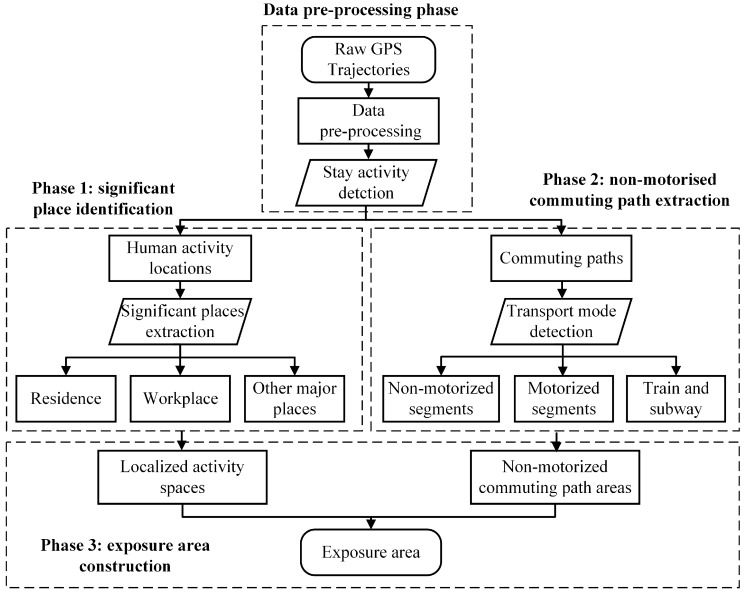
The methodology flowchart.

As shown in Figure 2, the proposed framework was composed of four phases, namely, the data pre-processing phase, significant places extraction phase, non-motorized commuting paths extraction, and exposure area construction phase.

### 3.1. Identification of Significant Places

Several criteria were developed to reduce the spatial drift and data insufficiency in the pre-processing stage. GPS trajectories or points that satisfied the following criteria were removed: (1) GPS points further than 500 m from their consecutive points; (2) GPS trajectories with less than one minutes traveling time; and, (3) GPS points located outside the study area.

#### 3.1.1. Step 1: Extraction of Activity Locations

**Figure 3:**
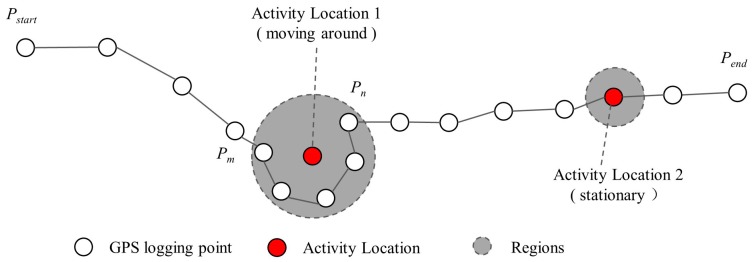
Extracting activity locations from GPS trajectory.

As a single GPS point has no semantic information, we first extracted human activity from the GPS trajectories, which was defined as a meaningful location where people spent their time [38]. As illustrated in Figure 3, two situations should be considered to extract human activities: one case was that an individual roamed around a geospatial region for a certain period like visiting a park; and the other situation was that a user stayed at a fixed location exceeding a time threshold, like entering a building [39]. Furthermore, it should be noted that for the latter case, there were some random movements in the GPS records due to the inaccuracy of the GPS loggers even if the participant did not move [40]. Therefore, a human activity could be detected if the sub-trajectory from point $p_{m}$ to point $p_{n}$ satisfied the following constraints:

(1) $$Dist\left(p_{m},\;p_{n}\right)<D_{max}\;\&\&\;Td\left(p_{m},\;p_{n}\right)>T_{min}$$

where $Dist\left(p_{m},\;p_{n}\right)$ refers to the Euclidean distance between $p_{m}$ and $p_{n}$. $Td\left(p_{m},\;p_{n}\right)$ refers to the timespan of the sub-trajectory. $D_{max}$ and $T_{min}$ are the two tuning parameters corresponding to the spatial range and dwelling time, respectively. In this study, we followed previous studies to use a distance threshold of 200 m and a time threshold of 20 min [41,42]. The centroid of the sub-trajectory—interpreted as a human activity—was used to represent the activity location [41]. In addition, the origins and destinations (ODs) of travels over one hour were also obtained due to their significance in mining individual activity patterns. As the individual stay activities are not bound to the street, in this stage, we did not match the activity locations to the street network.

#### 3.1.2. Step 2: Detection of Significant Place Candidates

**Figure 4:**
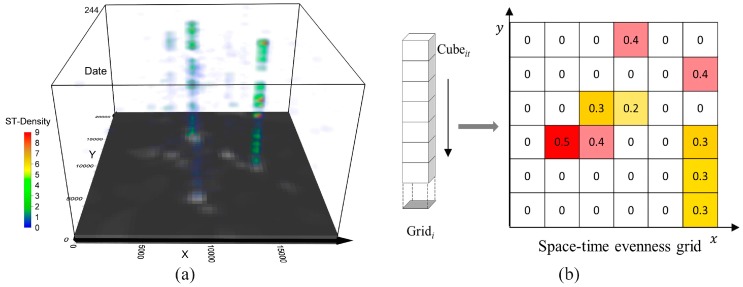
(**a**) An example of space-time kernel density estimation (ST-KDE) cube based on activity locations of #user 065. The base of the cube was the cumulative density by summing space-time density on each layer together. (**b**) Schematic diagram of compressing the space-time density cube into the space-time evenness grid.

In the next step, ST-KDE was integrated with DBSCAN to identify individual significant places according to the space-time proximity of the activity locations. By extending two-dimensional (2D) kernel density estimation to the three-dimensional (3D) form that accounts for time dimension, ST-KDE provides an efficient way to interpret the space-time patterns of point events and lifts our ability to reveal the spatio-temporal hotspots [43]. In this study, the input was the activity locations obtained in Step 1, and the output was a raster volume where each space-time cube C(x,y,t) was assigned a density estimation. For each cube, the space-time density $\hat{f}\left(x,y,t\right)$ was estimated using the following formula:

(2) $$\hat{f}\left(x,y,t\right)=\frac{1}{nb_{s}^{2}b_{t}}\overset{n}{\underset{i=1}{\sum }}k_{s}\left(\frac{x-x_{i}}{h_{s}},\frac{y-y_{i}}{h_{s}}\right)k_{t}\left(\frac{t-t_{i}}{h_{t}}\right)$$

where *n* is the number of the activity locations. $h_{s}$ and $h_{t}$ are the spatial and temporal bandwidths, respectively. $k_{s}$ and$\;k_{t}\;$are the space and time kernel functions to determine the weight of point $P_{i}\left(x_{i},y_{i},t_{i}\right)$ according to its space-time distance to the cube centroid P(x,y,t). In particular, the Epanecknikov kernel was utilized as a result of its good fitness to geographic phenomenon [44]. The space-time K-function algorithm proposed by Delmelle et al. (2011) was adopted to find the optimal bandwidths, and a pair of bandwidth (200 m, 1 day) was applied [45]. Figure 4a shows an example of ST-KDE cube based on user #065’s activity locations.

**Figure 5:**
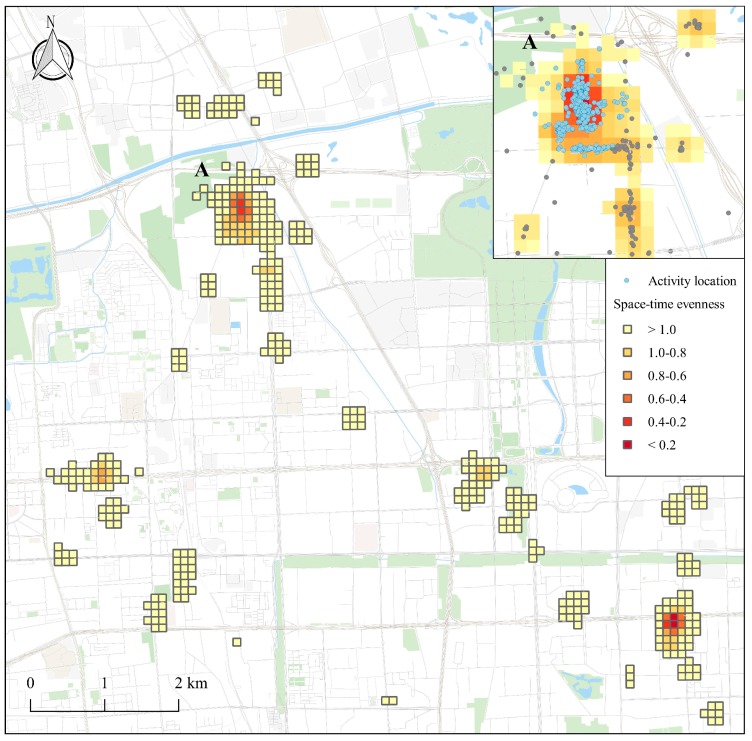
An example of space-time evenness grids. The dark color suggests uniform activity distribution across time and light color means intermittent occurrence of activities. Cluster A is a candidate of significant places.

To identify significant places from the long-term GPS dataset, we proposed a space-time evenness index (STEI) to measure the spatio-temporal dispersion of the activity locations by compressing the human activity intensities into a continuous surface (Figure 4b). For each cell $Grid_{i}$, we got the STEI value by calculating the coefficient of variation (CV) of all of the space-time density with the same spatial base. The formulas are:

(3) $$\bar{STD_{i}}=\frac{\sum_{t=1}^{T}STD_{it}}{T}$$

(4) $$\delta_{i}=\sqrt{\frac{\sum_{t=1}^{T}{\left(\bar{STD_{i}}-STD_{it}\right)}^{2}}{T}}$$

(5) $$STEI_{i}=\frac{\delta_{i}}{\bar{STD_{i}}}$$

where $\bar{STD_{i}}$ refers to the mean of cumulative space-time density on $Grid_{i}$ within the observed period T, and $STD_{it}$ refers to the space-time density for $Cube_{i}$ on day t. ${\mathrm{STEI}}_{i}$ refers to the variation of activity intensity on $Grid_{i}$, whereas the small value of STEI indicated an even temporal distribution of day-to-day activities, and a large value of STEI appeared when there existed only explosive visits in a short period of time. Therefore, the indicator could be employed to approximately detect the significant places from a long-time perspective. As the output of STEI was raster-based, which may give rise to the inaccuracy in detecting the significant place candidates, the hotspot areas were first extracted from the STEI surface and DBSCAN was further used to incorporate the evidence from space, as well as refining the spatial precision. Finally, the activity clusters intersected with or bounded by the hotspot areas in the space-time evenness surface were considered as the candidates of significant places. Figure 5 shows an example of the identification of significant places by combining the space-time evenness grid with the clustering result.

#### 3.1.3. Step 3: Labeling of Significant Places

To label the significant place candidates with semantic tags (e.g., residence and workplace), we first ranked the candidates of significant places by the frequency of reoccurrence and stay duration, then the use of places across the day were compared. The place where the individual spent the night (8:00 p.m.–6:00 a.m.) in most cases was marked as the residence and the workplace was identified when activities in a particular region demonstrated a dense distribution in working/school hours (8:00 a.m.–12:00 a.m. and 2:00 p.m.–6:00 p.m.) [46,47,48]. In short-period GPS studies, this method may introduce theoretically possible issues of people who work nightshift or those who had strong social ties to stay overnight with other families or friends [49]. To resolve the former issue, we compared the candidate of residences and workplaces with related POIs. For the latter issue, our method was based on the evidence from long-period observations and could filter out places that the participant had visited sparsely. Theoretically, some participants may spend most of their nighttime at others for a long period. In that case, these places played the role of home and were regarded as the residences. The candidate places except for the residences and workplaces were matched to the POIs dataset to determine their attributes, and these places were collectively termed as other major places.

### 3.2. Extraction of Non-Motorized Routes

GPS trajectories across the significant places were split into segments to extract the non-motorized commuting routes. Typically, a new segment was created if the time difference between two consecutive points was greater than five minutes [50]. To detect the transportation modes, the support vector machine (SVM) classification, which is a common approach to infer transportation modes from raw GPS trajectories, was adopted [51]. In this study, a linear kernel was chosen as the basis for the hyperplanes due to its short training time and feature transformation computation simplicity [52,53]. The SGD classifier, an efficient implementation based on a linear SVM, was then used to extract the non-motorized segments [35]. For each segment, four kinds of features, namely, speed, bearing change, distance, and duration were employed to construct the classifier [54]. The feature extraction of speed and bearing change was based on the cumulative statistics of the constituent GPS points rather than the features of the segment as the mean, minimum and maximum of these variables fail to reflect the actual distribution in many cases [49]. For instance, to obtain the speed features, a cumulative histogram was created to record the speed distributions (cumulative speed and the amount of time spent at them) and the speed where the cumulative value surpassed 10% to 90% of time were extracted. 

To train the classifier, the user-annotated transportation modes were aggregated into four categories: airplane, train, motorized mode and non-motorized mode based on the similarity of the movement features. The train label included data marked as the train and subway. The motorized mode was a combination of taxi, bus, and car. The non-motorized mode was composed of walk, run and bike. To validate the accuracy of the classification, one half of the annotated segments were randomly selected as the training samples and the others were used as the validation data. The results showed that the classification precision of non-motorized trips was up to 93%, which was a quite high value when compared with similar studies and could meet the study requirements [55]. Once the classifier was validated, we used it to classify the remaining dataset. Some post-processing steps were further employed to adjust the isolated segments surrounded by tracks labeled with different transportation modes and merge adjacent segments with the same transportation mode. Moreover, the motorized and non-motorized commuting paths across the significant places were matched to the road network using Graphhopper, respectively.

### 3.3. Construction of Exposure Area

Before describing the construction method of the exposure area, we have to define the localized activity spaces and the non-motorized path areas that are mentioned in this paper. In light of the overall activity space, the localized activity spaces (residential space, workspace and other major spaces) were defined as “the subset of locations visited by an individual over a given period, corresponding to her/his exhaustive spatial footprint around the anchor points (residence, workplace and other major places)”. This definition has been implicitly proposed by Chaix et al. (2012) to assess individual mobility patterns [24]. The non-motorized path areas were defined as “the subset of locations with which individuals have direct contact as the result of day-to-day non-motorized commuting behaviors” with reference to the daily (potential) path area [20,56].

To model the localized activity spaces, three kinds of geometry were created based on the notion of activity space and home range, such as network-based street buffer (NSB), SDE, and MCP [57]. NSB were created using the three most widely-used radii (200 m, 500 m and 1000 m) around the anchor points [58]. SDE and MCP were generated entirely based on the distribution of the activity location clusters marked as the residences, workplaces, and other major places. To determine a proper geographic representation for localized activity spaces, we evaluated the representativeness of these definitions in terms of the ability to capture clustered activities, the ability to filter out unclustered activities and the geometric area. Results indicated that MCP was superior to NSB and SDE in characterizing individual activities in the local scale (see Table S1 and Figure S1 in Supplementary Materials) and was used in later analysis. Furthermore, a 50 m buffer was further employed for the non-motorized trips to represent the participants’ exposure area along their commuting routes, as it could capture the food outlets that were accessible along the street [32]. The exposure area is composed of the residential space, workspace, other major spaces, and the non-motorized commuting path areas. Each part of the exposure area (e.g., residential space) has its spatio-temporal characteristics, such as the spatial extent, duration, and timing of individual activities.

### 3.4. Food Environmental Exposure Evaluation

To evaluate the foodscape exposure, the count of food outlets, the Physical Food Environment Indicator (PFEI) [59] and the diversity of average densities index (DADI) [60] were adopted to evaluate individual exposure to the food environment. PFEI is defined as the proportion of fast-food restaurants, convenience stores and small food stores (merged into convenience stores in this study) to all outlets in a certain region [59]. As fast-food restaurants and convenience stores are commonly classified as unhealthy (less healthy) outlets, PFEI reflects the healthiness of local food environment [61]. The value of the PFEI ranges from 0 to 1 and the higher the PFEI, the less healthy the food environment. The DADI is defined in the entropy form and is often used to calculate the diversity of local food services:

(6) $$DADI=-\overset{n}{\underset{i=1}{\sum }}p_{i}\times \frac{ln\left(p_{i}\right)}{\ln\left(n\right)}$$

where n is the number of the food store categories in the area. $p_{i}$ refers to the ratio of the i-th category food outlets. The value of DADI ranges from 0 to 1, where a higher level of the DADI indicated a more diverse foodscape.

To incorporate the temporal dimension into the food exposure assessment, a time-weighted contextual measure was introduced based on the stay duration an individual spent in multiple contexts (residential spaces, workspaces, other major spaces and the commuting path areas) [62]. The following formula was used to derive the time-weighted exposure measures:

(7) $$TWE_{i}=\overset{n}{\underset{j=1}{\sum }}D_{ij}\times W_{j}$$

where $TWE_{i}$ is the aggregated exposure to food services of the *i*-th category in multiple contexts. $D_{ij}$ refers to the count of food outlets of the *i*-th category in the *j*-th place. $W_{j}$ refers to the time weight for the *j*-th place, which is calculated as the ratio of the stay duration in the *j*-th place to the average stay duration in multiple contexts. Note that the time weight was determined strictly based on evidence from the entire observation, rather than a fragmentary period or empirical assumptions. The temporal constraints of food acquisition were further taken into account by matching the timing of individual activities and the opening hours of the food outlets. Only food outlets in their operating time when the individual activities took place were included in the assessment of food exposure.

## 4. Results

### 4.1. Description of the Study Sample

As shown in Table 1, the study samples (*n* = 107) were composed of full-time employees, government staff, college students and research fellows [36,41]. Young people were the main GPS data contributors and the average age of the participants was 24. The majority were between 22 and 30 years old, accounting for 75% of study samples, and people younger than 22 and older than 30 contributed 16% and 9% of the data, respectively. The participants were gender balanced and their education background ranged from undergraduate students to PhD holders.

**Figure 6:**
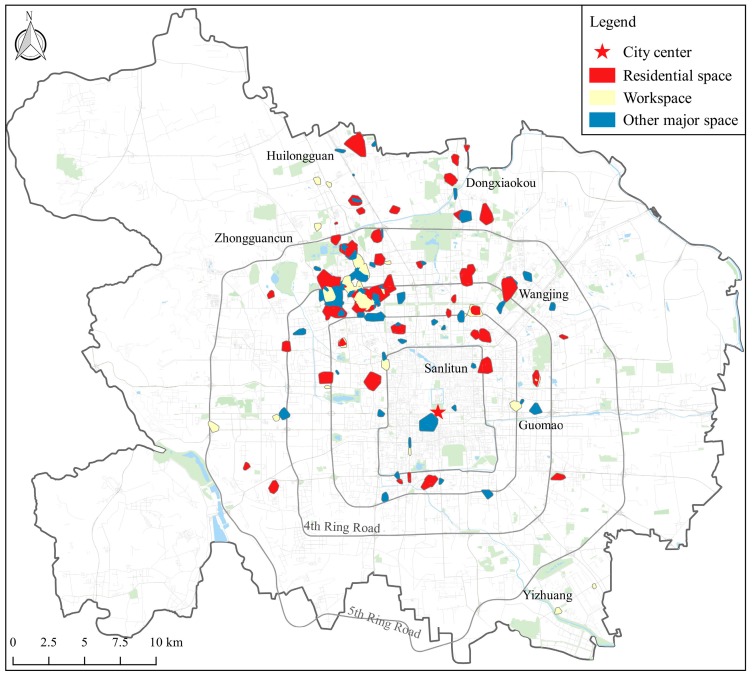
The aggregated distribution of the participants’ localized activity spaces (*n* = 107).

As shown in Figure 6, most of the participants’ localized activity spaces were distributed in the northern part of Beijing, especially in the area that is encompassed by the North 2nd Ring Road and North 5th Ring Road. Moreover, there were evident separations between the spatial distributions of different types of localized activity spaces. The residential spaces distributed dispersedly and the majority were situated in the outer zones (outside the 3rd Ring Road). A considerable number of participants had their residential spaces located in the new districts of urban development. In contrast, the workspaces were mainly aggregated in the industrial parks and office zones such as Zhongguancun and Yizhuang. Furthermore, the places where the individual regularly visited besides working and living were either close to their residences or workplaces, and a small part of isolated places arose at the commercial centers, like the Beijing Central Business District (Guomao), Wangjing and Sanlitun. These findings were consistent with related studies [41].

### 4.2. Analysis of Foodscape Exposure in Multiple Context

Table 2 shows the characteristics of the food outlet numbers among each part the exposure areas. In general, there were significant differences among the magnitude of food exposure in the residential spaces, workspaces, other major spaces, and the commuting path areas. The number of all the food outlets along the commuting routes (147.5) was 93% higher than that of the residence spaces (76.3), and the food outlet numbers in workspaces (54.0) and other major spaces (41.5) were lower than that of the residence spaces at percentages of 29% and 46%, respectively. This tendency was also tenable when taking the categories of food outlets into consideration. However, the gap of foodscape exposure among the localized activity spaces varied by food outlet types. For example, the greatest variations of food outlets numbers that the participants were exposed to around their homes and workplaces were found in restaurants and fast food outlets. As for the commuting routes and residential space, convenience stores, and vegetable and fruit stores dominated the differences.

**Figure 7:**
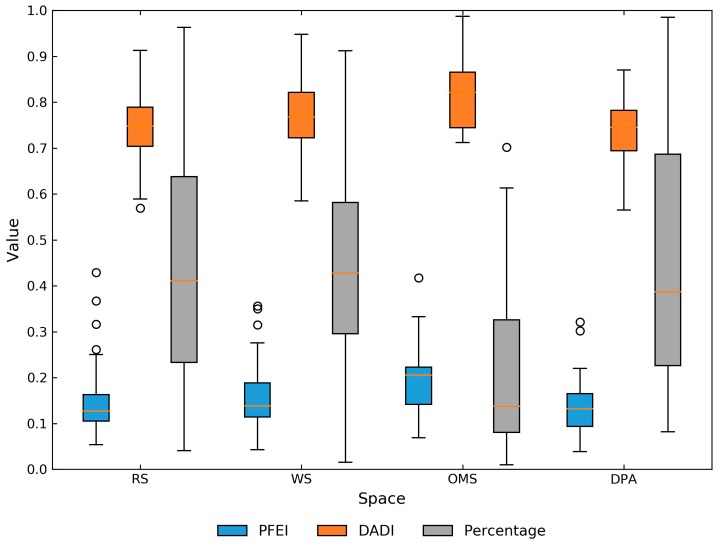
The PFEI, DADI, and Percentage of food outlet numbers in different spaces. (Abbreviations: RS-residential space, WS-workspace, OMS-other major space, DPA-non-motorized commuting path area).

Figure 7 shows the variations of PFEI, DADI, and the percentage of food outlet numbers in different spaces. On average, the lowest level of PFEI was found in the residential spaces and the commuting path areas (0.14), indicating that the participants encountered the healthiest foodscape around home and along the commuting paths, except the few participants who lived near the 2nd Ring Road. When compared with the residential spaces and the commuting path areas, the workspaces had a greater level of PFEI (0.16) and the largest PFEI was observed in other major spaces (0.21).

In addition, the diversity of the food environment in different spaces demonstrated similar patterns with PFEI: the largest DADI was found in other major spaces (0.82), followed by the workspaces (0.77), commuting path areas (0.75), and residential spaces (0.74). Furthermore, the percentage of food outlets in different parts of the exposure area demonstrated a broad range of variations (0.2–0.7), suggesting that there were great variations in the contribution of food exposure in different spaces. However, on the whole, the commuting path areas, residential spaces and workplaces made up the largest part of the foodscape in the participants’ daily life, and the ratio of food outlets in other major spaces was quite small.

Table 3 shows the correlation coefficients of food outlet numbers in the residential spaces related with the workspaces, other major spaces and commuting areas, using the Spearman-Rank analysis method. No significant correlations of food exposure between these places were observed, except for the convenience stores in the residential spaces and workspaces, where the relationship was weak (0.33). This result indicated that the foodscape in the residential spaces could hardly characterize the overall environment.

### 4.3. Analysis of Overall Foodscape Exposure

**Figure 8:**
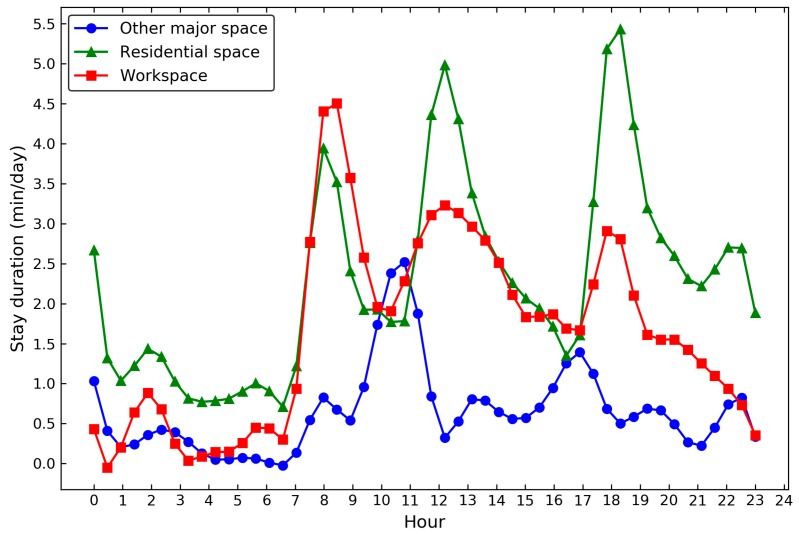
The timing of daily activities and average stay duration in different spaces per day (*n* = 107).

Figure 8 shows the time distribution of individual daily activities in different spaces. There were three activity peaks in the residential spaces during the day, namely, 7:00 a.m.–9:00 a.m., 12:00 p.m.–1:00 p.m., and 5:00 p.m.–8:00 p.m. Additionally, a less activity peak was found near midnight (10:00 p.m.–1:00 a.m.), which implied that the participants were more likely to interact with the food outlets around the residences during these periods. The workspace shared similar activity patterns with the residential space, but its magnitude was relatively smaller, except during rush hours (8:00 a.m.–10:00 a.m.). The leisure activities were flourishing during the valley period of home and work-related activities (10:00 a.m.–11:00 a.m. and 4:00 p.m.–5:00 p.m.).

**Figure 9:**
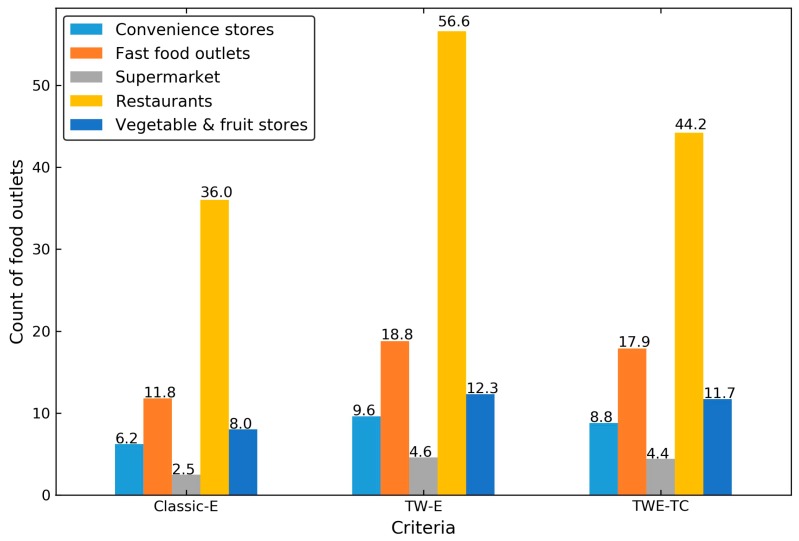
Average food outlet counts of the overall foodscape using Classic-E, TW-E, and TWE-TC, stratified by food outlet types.

Figure 9 shows the average food outlets numbers of the overall foodscape exposure. The classic exposure, or Classic-E, was the average of the food outlets numbers in the residential spaces, workspaces, other major spaces and non-motorized commuting path areas for each of the participants. The time-weighted exposure, or TW-E, was calculated as the cumulative sum of the product of the food outlets numbers in each space and the proportion of stay duration. The time-weighted exposure with temporal constraint, or TWE-TC, was the extension of TW-E by incorporating the timing of the activities and the opening hours of nearby food outlets. 

On average, the participants were exposed to 6.19 convenience stores, 11.75 fast food outlets, 2.49 supermarkets, 35.98 restaurants, and 7.99 vegetable and fruit stores in the spatial dimension. Taking the stay duration and temporal constraints into consideration, the number of food outlets changed disproportionately. A typical case was the supermarkets and restaurants based on the criteria of TW-E and TWE-TC, the number of restaurants declined from 56.60 to 44.17 when we matched the timing of individual activities and the opening status of restaurants the participants went by, whereas the number of supermarkets barely changed.

**Figure 10:**
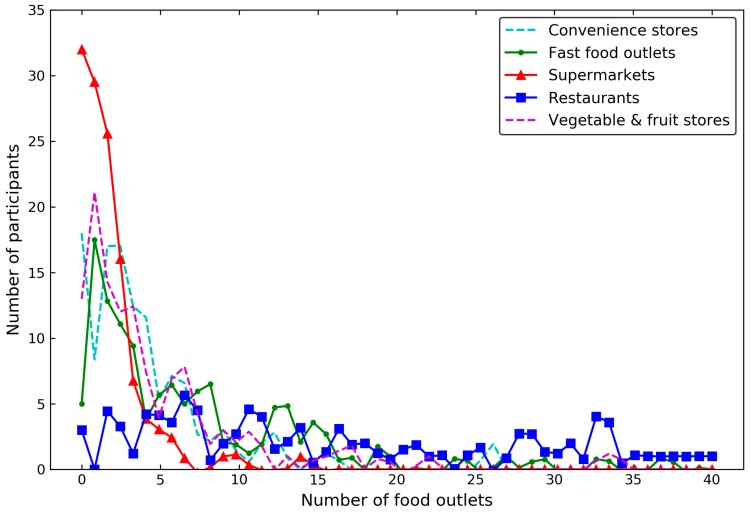
The overall food outlet distributions by food outlet types based on TWE-TC.

Figure 10 shows the density curves of the food outlets numbers in the overall environment by type, based on TWE-TC. The majority of the participants were exposed to a small number of supermarkets, convenience stores, vegetable and fruit stores, and the overall exposure to over 16 outlets of these types was rare. When compared with other kinds of food outlets, the restaurant distribution was broader and shifted to the right, indicating that there were great variations in the number of accessible restaurants among the participants. On the other hand, although numerous participants held wide-range exposure areas that contained a considerable number of food outlets that seemed to be accessible, the majority only witnessed a few food outlets when taking the stay duration and temporal constraints into consideration (Figure 6).

## 5. Discussion

### 5.1. Main Findings

As the pilot study showed, there were considerable variations in the magnitude of food outlets between the residential environment (residential space) and the non-residential environment (workspace, other major spaces and the commuting path areas) from the perspective of space (Table 2). When compared with non-residential context, the residential environment only contributed a small part to the overall foodscape exposure, more specifically, the food outlets numbers in the residential space were even smaller than that along the non-motorized commuting routes. In addition, the composition of the food outlets varied by spaces (Figure 7). In general, the percentage of fast food restaurants and convenience stores was the lowest in the residential space and the non-motorized commuting path areas. Other major spaces encountered the highest ratio of these two kind food outlets, followed by the workspace. Similar patterns were also found in the diversity of food outlets. Nevertheless, the quantity of food outlets in the residential space and other non-residential areas were poorly correlated. These findings indicated that the foodscape exposure was heterogeneous in different spaces and the foodscape exposure in the residential environment could hardly represent the overall foodscape that people encountered while engaged in their routine activities [7,11]. Perhaps this could explain why neighborhood effects based on the residences were often weak and even insignificant [63]. A different finding from prior neighborhood-based literature was the difference of the quantity of food outlets between the residence and workplace, where the number of food outlets in the workspace was greater than that in the residential space [64,65]. This was partly because the participants’ localized activity spaces were individual-specific and the size of workspaces was smaller than that of the residential spaces in many cases (see Table S2 in Supplementary Materials), while in the literature, the same sized neighborhoods were assigned to the home and workplace [8].

The variations of foodscape exposure in different spaces may be related to the jobs-housing separations in the area [66]. The demographic census in 2013 showed that 51.1% of the resident population in Beijing were gathered outside the 5th Ring Road, whereas 70% of the residents were employed inside the 4th Ring Road [67]. Similar patterns were found here: as shown in Figure 6, numerous participants lived in the outer areas and worked in core areas. Additionally, the structure of foodscape in these places varied greatly. For example, other major spaces where the participants conducted their leisure activities were mainly distributed in the major business centers of the city and the commercial streets, so that the variety of the food outlets and the proportion of fast food restaurants were comparatively higher. In contrast, the residential spaces were mainly located near the residential communities and the proportion of fast food restaurants was smaller. Moreover, the jobs-housing separations gave rise to the long-distance commuting journeys and indirectly provided the opportunity for individuals to interact with the foodscape outside their residences and workplaces.

Taking stay duration and temporal constraints into consideration, most of the participants were exposed to only a small number of food outlets during their routine activities. Restaurants were the main difference in the overall foodscape exposure in the study sample. Although the participants encountered a larger number of food outlets along the commuting routes, the commuting time was shorter when compared with their stay durations in the residential spaces and workspaces, so that their contribution decreased correspondingly (see Table S2 in Supplementary Materials). These findings suggested that relying solely on the spatial dimension would likely lead to the mischaracterization of the overall foodscape exposure and therefore attenuated the associations between exposures and health outcomes [22]. In addition, when incorporating the timing of individual activities and the operating hours of the food services, the number of food outlets that were accessible changed disproportionately by type. On average, the number of restaurants and vegetable and fruit stores decreased by 22% and 8%, respectively, while the number of supermarkets only declined by 4%. This phenomenon was probably related to the way of the individual life and the regularity of the business. For example, if a full-time worker started off early in the morning and returned home late in the evening, he/she might need to purchase food on the way to work or home. In this period, many restaurants did not operate (especially for those that did not provide breakfast or closed early in the evening), whilst most supermarkets were open, thereby shaping the foodscape dynamically.

### 5.2. Strengths and Limitations

One major highlight of the proposed framework was that the proposed exposure area could capture the non-residential environment experienced by the individuals during their daily movement. The size of the exposure area was individual-specific and was determined by the distribution of individual daily activities. This differs from conventional place-based methods where the exposure area was mostly defined as a static neighborhood [7]. In this study, the proposed framework constructed the exposure area by integrating the local activity spaces (e.g., residential spaces and workspaces) and the commuting path areas. Furthermore, the commuting path areas were created entirely based on non-motorized commuting routes rather than the entire trips that involved a mix of transportation modes. This is a step toward the refinement of measuring the foodscape exposure and may potentially reduce the uncertainty of modeling the interaction between the individual and the food environment along the commuting paths [28].

Another strength of the proposed framework was that it enabled us to incorporate the space-time heterogeneity (spatial extent, stay duration, and timing) of individual behaviors in different spaces and the dynamics of the surrounding food environment. This is particularly important as the contextual exposure varies by places and time, whilst individual activities around different daily life centers (e.g., home and workplace) had various space-time patterns (Figure 8). Moreover, the adoption of long-term GPS data was superior to the short-period data in understanding the persistence of human activity patterns over time, and helped to differentiate the significant places visited at a high frequency and journeys from those that were rarely visited. This is a further exploration on addressing the uncertain geographic context problem (UGCoP) caused by the overall activity space, where the frequency, duration and temporality of individual daily activities were overlooked [27,68].

There were, however, several limitations in this study that can be attributed to the data quality and study design. First of all, the proposed method was procedure-oriented, and in some phases, parameters were needed (e.g., the space-time thresholds to define a human activity, the parameter to define activity clusters and the distance to define the commuting path areas). Although there have been numerous approaches specializing in the parameter selections that can be inferred to, simple combinations of these methods designed for different purposes may not always find the appropriate parameters, which would more or less bring about uncertainty in the construction of the exposure area.

Secondly, the adoption of stay duration in measuring multiple contexts of food exposure also needs reconsideration. Although the use of time-weighted exposure measure was commonsense in environmental studies, using the cumulative sum of food outlets numbers multiplied by the time-weight to measure the overall exposure remains to be verified [62]. More studies to further our understanding of the relationship between cumulative foodscape exposure and the stay duration are expected.

Another limitation to this study was related to the data quality of the dataset. The small samples limited the power to reveal the differences of foodscape among different spaces, and the restricted demographic scopes of the sample limited the generalizability of the findings. For example, due to the clustering of the participants in space, overlaps of the residential spaces and workspaces were found in the study sample, and therefore raised concerns about the spatial autocorrelations. The introduction of geographically weighted regression and spatial econometric approaches may resolve this issue. Nevertheless, as the POI dataset in 2012 was the only data accessible, information about the food outlets (e.g., operating status and opening hours) were not in step with individual activities timely. All of these issues should be considered in the next step.

## 6. Conclusions

This study explored the use of long-term GPS data in investigating individual foodscape exposure. To derive the exposure area, a novel framework based on the space-time proximity of individual physical activities was proposed to extract the localized activity spaces around daily life centers and the non-motorized commuting routes. When compared with conventional methods, the newly proposed exposure areas were individual-specific and could incorporate the internal heterogeneity of individual activities (spatial extent, stay duration, and timing) and the dynamic of the context. The pilot study in Beijing suggested that there were significant variations in the magnitude as well as the composition of food outlets in the exposure area, and the cumulative foodscape exposure far outweighed the food exposure that experienced by the individual in the residential space alone. Furthermore, restaurants were the main differences of the overall foodscape exposure among the participants. In the future, we will improve the robustness of the proposed method and implement our framework into a dynamic model for more researchers to use. Spatio-temporal clustering and other classification methods will be involved in processing the long-term GPS datasets. In addition, if data are available, much deeper research will be conducted to reveal the relationship between public health and the foodscape exposure accounting for the internal heterogeneity of individual movements and the temporal dynamics of the food environment.

## Figures and Tables

**Table 1:** Descriptive statistics of sample characteristics (*n* = 107).

Characteristic	Percentage	*n*
Age		
≥30	9%	10
26–29	30%	32
22–25	45%	48
≤22	16%	17
Gender		
Male	54%	58
Female	46%	49
Career		
MSRA employees	18%	19
Employees of other companies	14%	15
Government staff	10%	11
College students and Researchers	58%	62

**Table 2:** Descriptive statistics of food outlets counts in residential space, workspaces, other major spaces, and commuting path areas (*n* = 107).

Category		RS	WS	Difference at WS	OMS	Difference at OMS	DPA ^a^	Difference at DPA ^a^
All outlets	Mean (SD)	76.3 (139.2)	54.0 (53.1)	−29% *	41.5 (47.3)	−46% *	147.5 (372.9)	+93%
	Range	292	282		245		379	
Convenience stores	Mean (SD)	6.5 (10.7)	5.2 (5.3)	−21% *	3.8 (3.9)	−42% *	15.3 (43.8)	+136% *
	Range	74	24		20		159	
Fast food outlets	Mean (SD)	14.9 (29.9)	10.4 (11.5)	−30%	7.5 (9.9)	−50%	25.2 (62.4)	+69%
	Range	116	65		67		114	
Supermarket	Mean (SD)	2.9 (5.0)	2.6 (2.4)	−19%	2.3 (2.6)	−30% *	5.2 (13.1)	+80% *
	Range	29	9		12		73	
Restaurants	Mean (SD)	43.0 (75.6)	29.5 (28.6)	−32%	22.9 (26.3)	−47%	82.9 (208.0)	+93%
	Range	198	139		131		427	
Vegetable and fruit stores	Mean (SD)	9.0 (20.9)	6.7 (8.2)	−25% *	5.0 (7.0)	−44% *	18.9 (48.6)	+110% *
	Range	41	45		32		104	

Note: * Significant difference (ANOVA, *p* < 0.05) between the food outlets numbers in residential space and other localized activity spaces. ^a^ Food exposure of DPA were defined as the sum of food outlets counted along the non-motorized commuting path areas. Abbreviations: RS-residential space, WS-workspace, OMS-other major space, DPA-non-motorized commuting path area.

**Table 3:** Correlation of food outlet counts in residential space (RS), workspace (WS), other major space (OMS), and the non-motorized commuting path area (DPA) (*n* = 107).

Food Outlet Type	RS × WS	RS × OMS	RS × DPA
Convenience stores	–0.02	0.33 **	0.10
Fast food outlets	0.10	0.09	0.01
Supermarket	–0.10	0.21	0.08
Restaurants	0.11	0.04	–0.04
Vegetable & fruit stores	–0.10	–0.05	–0.05
All food outlets	0.06	0.07	–0.02

Note: ** *p* < 0.01.

## Supplementary Materials

The following are available in the http://www.mdpi.com/1660-4601/15/3/405/s1. Figure S1: Results of different representation methods of localized activity spaces. Table S1: A summary evaluation of representativeness of the localized activity spaces. Table S2: Spatial extent, average stay duration, and frequency of stay activities around the anchor places and along the commuting routes (*n* = 107).
