# Supplementary material for: Using Individual GPS Trajectories to Explore Foodscape Exposure: A Case Study in Beijing Metropolitan Area
Source: Int J Environ Res Public Health. 2018 Feb 27;15(3):405. doi: 10.3390/ijerph15030405 (PMC5876950; doi:10.3390/ijerph15030405)
Supplement: Supplementary file 1 [file ijerph-15-00405-s001.docx]

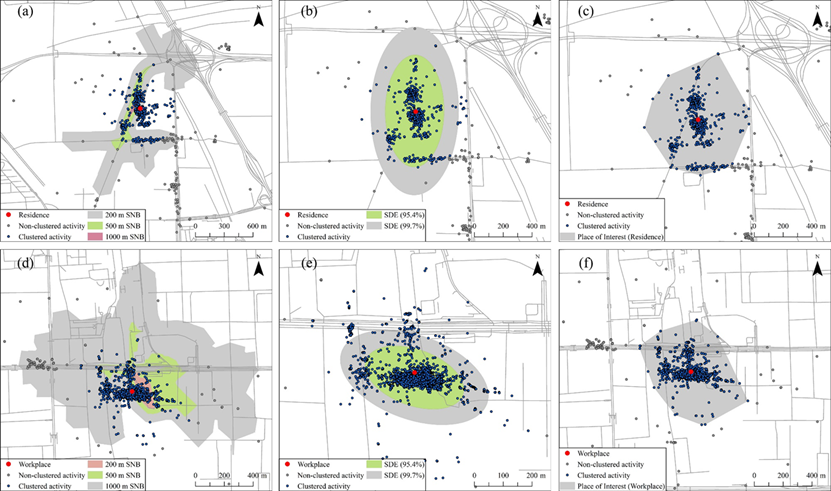


**Figure S1.** Results of different representation methods of localized activity spaces (localized SNB, SDE, MCP from left to right) corresponding to two typical urban structure: a sparse street network (**a**), (**b**), (**c**) and a dense street network (**d**), (**e**), (**f**).

**Table S1.** A summary evaluation of representativeness of the localized activity spaces.

| **Geographical Representation** | **Ratio of Clustered Activity Locations** | **Ratio of Non-Clustered Activity Locations** | **Area (km^2^)** | **Geometry Restrictions** |
| --- | --- | --- | --- | --- |
| 200 m Street network buffer | 11.3% | 0.0% | 0.02 | Street network |
| 500 m Street network buffer | 36.7% | 5.3% | 0.15 | Street network |
| 1000 m Street network buffer | 86.8% | 21.0% | 1.17 | Street network |
| Standard deviation ellipse (95%) | 95.4% | 0.0% | 0.05 | Data & Ellipse |
| Standard deviation ellipse (99%) | 99.7% | 0.2% | 0.42 | Data & Ellipse |
| Local minimum convex polygon | 100.0% | 0.0% | 0.40 | Data |

**Table S2.** Spatial extent, average stay duration and frequency of stay activities around the anchor places and along the commuting routes (*n* = 107).

|  | **RS** | | **WS** | | **OMS** | | **DPA ^a^** | |
| --- | --- | --- | --- | --- | --- | --- | --- | --- |
|  | **Mean (SD)** | **Range** | **Mean(SD)** | **Range** | **Mean(SD)** | **Range** | **Mean(SD)** | **Range** |
| Area (km^2^) | 0.5 (0.9) | 0.1–3.6 | 0.3 (0.2) | 0.1–1.1 | 0.2 (0.3) | 0.1–1.4 | 0.2 (0.3) | 0.1–1.2 |
| Perimeter (km) | 2.7 (1.9) | 0.4–7.5 | 2.1(0.9) | 0.6–4.6 | 1.9 (1.1) | 0.6–4.4 | 4.9 (6.1) | 0.3–9.9 |
| Duration ^b^ (min/day) | 55.4 (77.3) | 20.7–419.3 | 38.7 (52.9) | 0.0–263.6 | 15.8 (26.9) | 0.0–124.1 | 17.0 (24.8) | 0.6–122.6 |
| Activities ^c^ (times/day) | 1.8 (1.3) | 0.2–6.2 | 1.3 (0.7) | 0.2–3.7 | 0.6 (0.6) | 0.1–2.6 | 1.4 (2.1) | - |

Note: ^a^ Food exposure of DPA were defined as the sum of food outlets count along the commuting routes between activity spaces, accounting for transport modes. ^b^ Calculated as the average time people spent around the residences, workplaces, other anchor places and along the non-motorised commuting paths per day. The calculation was strictly based on the GPS trajectories, without estimating of people’s stay duration indoors that were not recorded. ^c^ Calculated as the count of stay activities around the anchor places per day. Abbreviations: RS-residential space, WS-workspace, OMS-other major space, DPA-non-motorized commuting path area.
